# Lipid Transporter Activity-Related Genetic Polymorphisms Are Associated With Steroid-Induced Osteonecrosis of the Femoral Head: An Updated Meta-Analysis Based on the GRADE Guidelines

**DOI:** 10.3389/fphys.2018.01684

**Published:** 2018-12-03

**Authors:** Xiantao Chen, Leilei Zhang, Dawei Liang, Jing Li, Fenzhi Liu, Hongxia Ma

**Affiliations:** ^1^Department of Osteonecrosis of the Femoral Head, Luoyang Orthopedic Hospital of Henan Province, Luoyang, China; ^2^Department of Osteoarthritis, Luoyang Orthopedic Hospital of Henan Province, Luoyang, China

**Keywords:** osteonecrosis, steroids, glucocorticoid, genetic polymorphism, meta-analysis

## Abstract

**Aims:** The purpose of this study was to assess the relationship between genetic variants and steroid-induced osteonecrosis of the femoral head (SONFH) in steroid use populations.

**Methods:** We searched the public databases up to April 15, 2018. This study analyzed only the single-nucleotide polymorphisms (SNPs) that have appeared in more than three studies and assessed the level of evidence by classifying the outcomes according to the Grading of Recommendations Assessment, Development, and Evaluation (GRADE) approach.

**Results:** The ABCB1 rs1045642 C>T mutation had a protective effect against SONFH in the allelic model (*I*^2^ = 50.2%; OR: 0.74; 95% CI: 0.55–1.00; *p* = 0.046). The rs2032582 mutation in the ABCB1 gene showed no relationship to SONFH (allelic model: *I*^2^ = 63.4%; OR: 0.85; 95% CI: 0.58–1.23; *p* = 0.382). In ApoB rs693, four models showed that mutations can increase SONFH risk, but the allelic model did not. The ApoB rs1042031 mutation increased SONFH risk in the dominant model (*I*^2^ = 50.3%; OR: 2.90; 95% CI: 1.49–5.66; *p* = 0.002).

**Conclusion:** An allelic model of ABCB1 rs1045642 showed that mutations have a protective effect against SONFH at a very low level of evidence. The mutations in ApoB rs693 and rs1042031 increase the SONFH risk with moderate levels of evidence.

## Introduction

Osteonecrosis of the femoral head (ONFH) is caused by the obstruction or interruption of local blood supply, resulting in tissue ischemia, necrosis, and finally bone collapse (Kim et al., 2011). The core decompression method only relieved the pressure of the pulp cavity and delayed the progression of necrosis (Yu et al., 2018). At present, long-term and/or large-dose use of steroids have become the most important risk factors for non-traumatic ONFH (Shigemura et al., 2011). The pathogenesis of steroid-induced ONFH (SONFH) is not yet clear and may be related to an imbalance in lipid metabolism and abnormal microcirculation (Johnson et al., 2004). The abnormal blood supply leads to apoptosis of osteocytes and osteoblasts, which causes bone loss and reduces bone mineral density (Luo et al., 2018). In addition, disordered lipid metabolism is another crucial pathogenesis that leads to increases in circulating lipid levels, microvascular fat embolisms, and lipid accumulation in the pulp cavity and ultimately causes osteocyte death.

Genetic polymorphisms have been found to be related to SONFH due to individual differences. Studies exploring the association between single-nucleotide polymorphisms (SNPs) and SONFH will help to identify the high SONFH risk population who use steroids. For these patients, it is necessary to avoid steroid application or to change the application strategy, increase the frequency of detection of SONFH, and enable early intervention. Furthermore, genetic variant-related studies can help to understand the pathogenesis of SONFH. Presently, several meta-analyses have been published that indicate that *PAI-1* rs1799768 (Gong et al., 2013), ABCB1 rs1045642 (Gong et al., 2013; Li et al., 2014; Zhou et al., 2015), and CYP3A variants (Guo and Deng, 2017) are related to the occurrence of SONFH. However, there is still a dispute over ABCB1 rs2032582 (Gong et al., 2013; Li et al., 2014; Zhou et al., 2015). This study will explicitly exclude studies in which the control group includes a healthy population or other types of ONFH; only comparison studies between ONFH and non-ONFH in the steroid use population are included. Because all studies are based on case-control and cohort designs with low evidence levels, we also used the Grading of Recommendations Assessment, Development, and Evaluation (GRADE) guidelines to assess the outcomes (Guyatt et al., 2008). Overall, the relationship between genetic variants and steroid-induced osteonecrosis of the femoral head in steroid use populations will be assessed in this study.

## Methods

This meta-analysis was performed in accordance with the Preferred Reporting Items for Systematic Reviews (PRISMA) guidelines (Moher et al., 2009).

### Data source and search strategy

Two authors independently performed a literature search using PubMed, Embase, the Cochrane Library, and the Chinese public databases, including the China National Knowledge Infrastructure (CNKI), the China Biology Medicine (CBM) Database, the China Science Periodical Database (CSPD, Wanfang Database), and the VIP Journal Integration Platform (VJIP). Searches were performed for studies published up to April 15, 2018. The following terms were used in the search strategy: hormone, glucocorticoid, steroid, corticosteroid, osteonecrosis, necrosis, femoral, femur, femoris, whirlbone, polymorphism, SNP, genetic, mutation, genotype, allele, allelic, and variation. We also conducted manual searches of the reference lists of relevant reviews to avoid omissions.

### Selection criteria

The following studies were included in our meta-analysis if they fulfilled the inclusion criteria: (1), study has a case-control or cohort study design; (2), steroid-using patients are included; (3), study compares ONFH and non-ONFH patients after steroid use; (4), study assesses the association between SNPs and SONFH; and (5), study indicates the frequencies of specific alleles or the effect sizes of individual genotypes between cases and controls.

The exclusion criteria included the following: (1), study compares patients with SONFH to healthy populations or populations with other types of ONFH; (2), study is about family heredity; (3), study is not SNP-related; and (4), study does not report data pertaining to allelic frequencies or calculable effect size. In addition, conference reports, editor comments, reviews, and academic dissertations were also excluded from the analysis.

### Data extraction and quality assessment

Two authors independently extracted the following information from each eligible study: the first author's name, publication year, research location, sample size, average age, primary disease, diagnostic mode, and genes of interest. The methodological quality of the included studies was evaluated using the Newcastle-Ottawa Scale (NOS), a validated tool for evaluating the quality of observation studies that includes the following 3 subscales: selection, comparability, and exposure (Stang, 2010). We assessed all results for the level of evidence using the GRADE approach (Andrews et al., 2013), the average NOS score, and the number of patients included and then presented the data in a three-dimensional Manhattan plot.

### Statistical analysis

The Hardy-Weinberg equilibrium (HWE) of the included populations was calculated to assess the consistencies of allele frequencies between generations (Chen and Chatterjee, 2007). Association analysis was performed using five genetic models (Areeshi et al., 2013). The effect size was estimated by calculating the summary odds ratio (OR) and its 95% confidence intervals (CIs). The *I*^2^ statistic was used to estimate the degree of heterogeneity among the studies. If the *I*^2^ ≥ 50% (Q test, *p* < 0.1), a random-effect model was used; otherwise, a fixed-effect model was used. Subgroup analysis was also performed according to the ethnicity and location of the population. We assessed publication bias using the Begg and Egger tests. All tests were two-tailed, and a *p-*value of less than 0.05 was deemed statistically significant. We analyzed the data using the R program (Version 3.3.1) and STATA (Version 14.0).

We used GRADE to assess the level of evidence with four levels graded from high (best) to very low (worst). All objective studies used an observational study design, which downgrades the quality of evidence. Inconsistency was evaluated using the *I*^2^ and *p-*values of the Q test, and the outcome was imprecise if the standard error (SE) was larger than 0.2. Publication bias (reporting bias) also reduced the level of evidence. Finally, a large (OR > 2 or <0.5) or very large (OR > 5 or <0.2) effect upgraded the quality of evidence.

## Results

**Figure 1:**
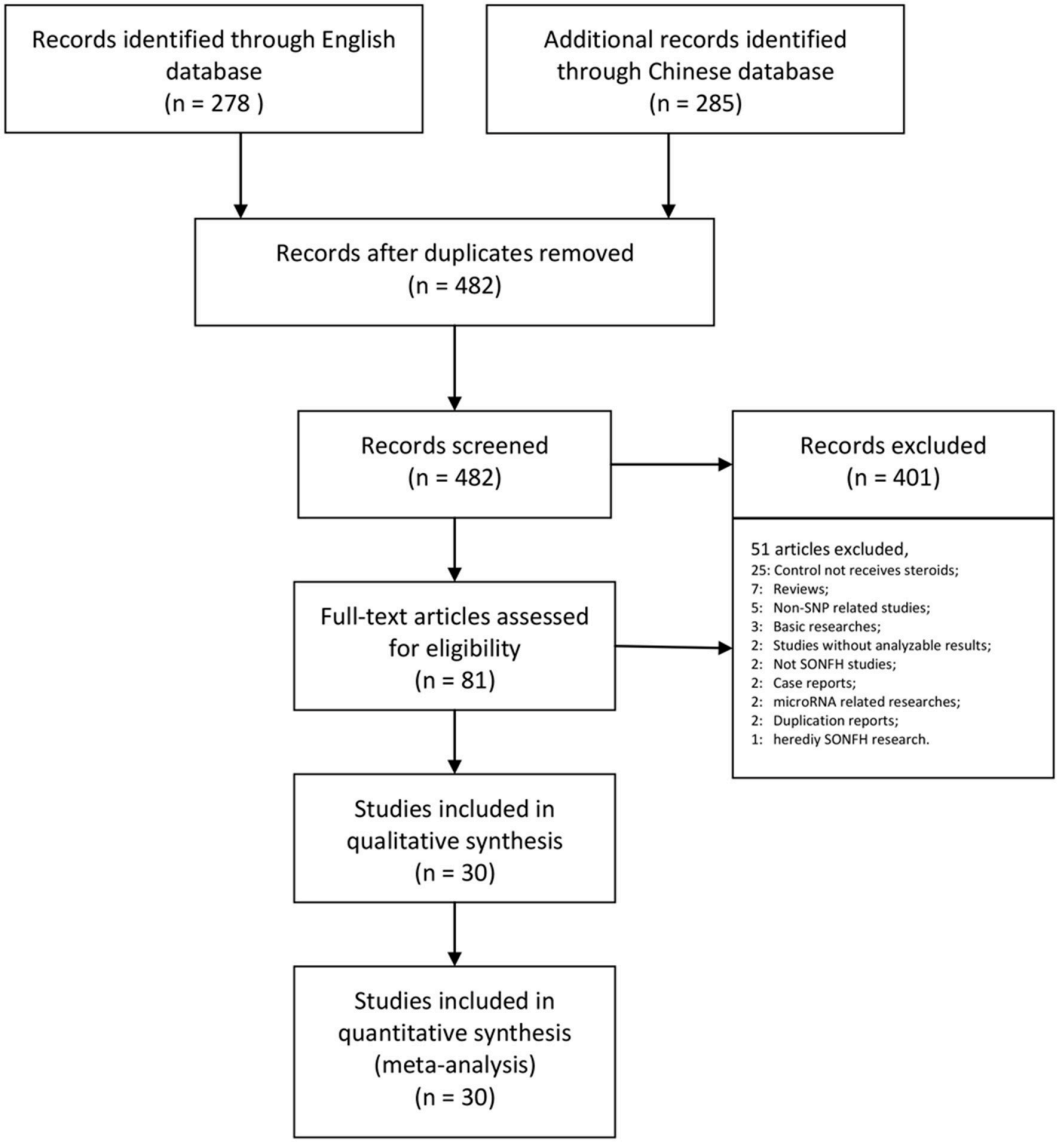
PRISMA flow chart illustrating the selection process of the studies included in our analysis. SNP, single-nucleotide polymorphism; SONFH, steroid-induced osteonecrosis of the femoral head.

Our research returned 278 English articles and 285 Chinese articles after removing duplications. After screening the titles and abstracts, 482 of these articles were excluded. The full texts of 81 articles were assessed, among which the control group did not receive steroid therapy (25); the article was a review (7); the study was non-SNP-related (5); the research was basic (3); the study did not report frequencies or effect size results (2); the study did not include steroid-related ONFH (2); the study was a case report (2); the research was microRNA related (2); the report was a duplicate (2); and the research was of heredity SONFH (1). Finally, we collected 30 trials assessing 7,553 patients for our meta-analysis (Table 1, Figure 1).

**Table 1:** Characteristics of the included studies.

**References**	**Local**	**Sample size**	**Average age^#^**	**Type of steroids**	**Primary disease**	**Genes**	**NOS score**
Zhao et al., 2017	China	193 (78/115)	40 (18–48)	Prednisone	SLE; ALL; RT; LT; NS; RA; AS	*GRG*	9
Plesa et al., 2017	Caucasian	304 (32/272)	NA	Prednisone	ALL	*BCL2L11*	9
Karol et al., 2015	Multinational	2955 (400/2555)	NA	Prednisone; dexamethasone	ALL etc.	GWAS	7
Wei et al., 2015	China	75 (45/30)	39 ± 10	Prednisone	SLE; NS; ENT; dermatologic disease	*ApoA1; ApoB; ApoE*	8
Zhang et al., 2014	China	200 (94/106)	44.5 (18–82)	Prednisolone	Hematologic; dermatologic; renal; ophthalmopathy; respiratory disease, etc.	*ABCB1*	7
Xue et al., 2014	China	322 (105/217)	39 (18–48)	Prednisone	SLE; ALL; OT	*ABCB1*	9
Cui et al., 2014	China	424 (223/201)	42.27 ± 15.71	Prednisolone	NA	*ApoA5*	7
Zeng et al., 2014	China	206 (108/98)	40 ± 10	Prednisone	SLE; NS; RT; Purpura; ENT; dermatologic disease	*ApoB*	8
Zhang et al., 2013	China	200 (94/106)	44.5 (18–82)	Prednisolone	Hematologic; dermatologic; renal; ophthalmopathy; respiratory disease, etc.	*PAI-1*	7
Wang et al., 2013	China	200 (94/106)	44.5 (18–82)	Prednisolone	Hematologic; dermatologic; renal; ophthalmopathy; respiratory disease, etc.	*PON-1*	6
Li et al., 2012	China	123 (70/53)	29 (18–73)	Prednisone	NA	*ABCB1*	6
Wei, 2011a	China	134 (63/71)	35.17 ± 11.73	Prednisone	NA	*ApoB; CYP1A2*	8
Wei, 2011b	China	134 (63/71)	35.17 ± 11.73	Prednisone	NA	*Factor V; GR; CBP; ApoB; CYP1A2*	8
Bond et al., 2011	UK	110 (43/67)	NA	Dexamethasone	ALL	*PAI-1*	8
He and Li, 2009a	China	48 (31/17)	32 (12–59)	NA	Hemoglobinopathies	*CYP3A4; ABCB1*	7
He and Li, 2009b	China	48 (31/17)	18–60	Prednisone	SLE; RA; psoriasis; nephropathy; desmosis, etc.	*CYP3A4*	7
Kuribayashi et al., 2008	Japan	157 (34/123)	35 (9–64)	Methylprednisolone; prednisolone	RT	*ABCB1; ApoB; CBP*	9
French et al., 2008	USA	361 (51/310)	NA (10–20)	Prednisone; dexamethasone	ALL	*ABCB1; PAI-1* et al. 11 Genes^##^	7
Wang et al., 2008	China	53 (16/37)	35 (16–78)	Methylprednisolone	RT	*TNF-α*	8
Tamura et al., 2007	Japan	157 (34/123)	35 (9–64)	Methylprednisolone; prednisolone	RT	*GR; CYP3A4; CBP; NCoA2*	9
Yang and Xu, 2007	China	127 (21/106)	34 (11–67)	Methylprednisolone; prednisone	SLE	*ABCB1*	9
Hirata et al., 2007a	Japan	112 (20/92)	NA	Methylprednisolone; prednisone	SLE	*ApoA*	7
Hirata et al., 2007b	Japan	158 (34/124)	36.1 (9–64)	Methylprednisolone; prednisolone	RT	*ApoB*	7
Ekmekci et al., 2006	Turkey	57 (19/38)	34.2 ± 9.3	NA	RT	*Factor V; Prothrombin*	7
Celik et al., 2006	Turkey	50 (11/39)	41 ± 11.79	Prednisolone	RT	*Factor V; Prothrombin; MTHFR*	8
Relling et al., 2004	USA	64 (25/39)	8.6 (2.7–18.8)	Prednisone	ALL	*MDR1(ABCB1)* et al. 13 Genes^###^	8
Asano et al., 2004	Japan	137 (31/106)	36 (9–63)	Methylprednisolone; prednisolone	RT	*PAI-1; MTHFR*	8
Asano et al., 2003a	Japan	80 (26/54)	NA	NA	RT	*CYP3A4; CYP2D6; CYP2C19*	7
Asano et al., 2003b	Japan	136 (30/106)	35.5 (9–63)	Methylprednisolone; prednisolone	RT	*ABCB1*	8
Ferrari et al., 2002	Switzerland	228 (26/202)	50 ± 12	Prednisone	RT	*PAI-1*	8

*ALL, Acute lymphoblastic leukemia; AS, Ankylosing spondylitis; ENT, Ear, nose, and throat; GWAS, Genome-wide association study; LT, Liver transplant; NA, Not available; NOS, Newcastle-Ottawa Scale; NS, Nephrotic syndrome; OT, Organ transplant; RA, Rheumatic arthritis; RT, Renal transplant; SLE, Systemic lupus erythematosus*.

# *: Mean ± Standard deviation; Mean/Median (Minimum-Maximum)*.

## *: TYMS; VDR; BGLAP; ESR1; LRP5; MTHFR; PAI-1; ABCB1(MDR1); PTH; PTHR; ACP5*.

### *: CYP3A4; CYP3A5; TPMT; UGT1A1; TYMS; GSTT1; GSTM1; RFC; MTHFR; GRG(NR3C1); MDR1(ABCB1); VDR; GSTP1*.

All studies used a case-control design except one that used a cohort design (Karol et al., 2015). In addition, Weibao Fang's two studies (Wei, 2011a,b), and Kuribayashi and Tamura's studies (Tamura et al., 2007; Kuribayashi et al., 2008) included the same patient population but did not assess the same SNPs. We analyzed different SNP results and excluded duplicated results. The NOS scores were six to nine points, and the overall quality was ideal in observational studies (Table 1).

**Figure 2:**
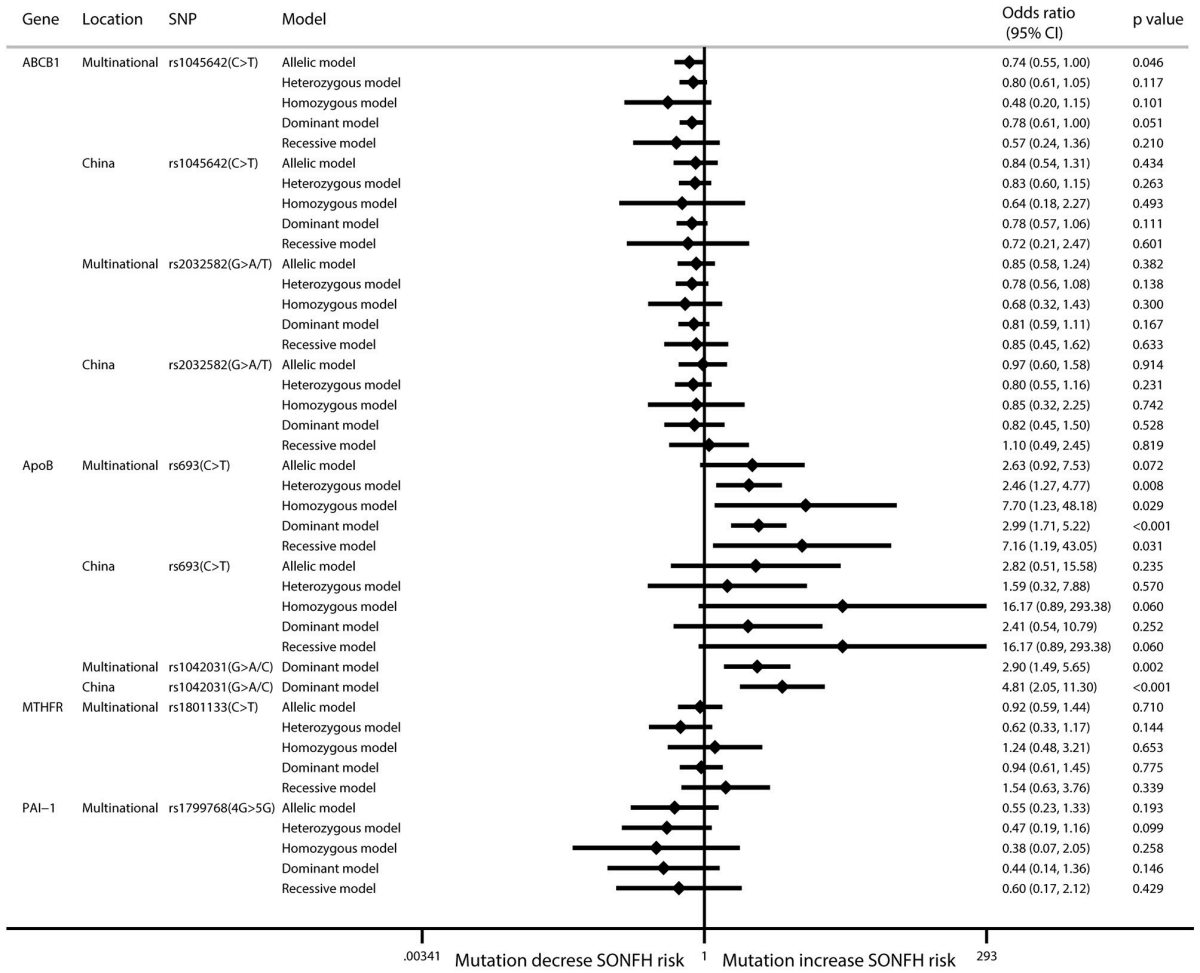
Forest plot of associations between mutations and SONFH risk. SNP, single-nucleotide polymorphism; SONFH, steroid-induced osteonecrosis of the femoral head.

Six SNPs in four genes were included in the meta-analysis. In the ABCB1 gene, rs1045642 is also known as C3435T, is located in the coding region, and is a synonymous mutation. In this research, the pooled results for rs1045642 showed that the C > T mutation protected against SONFH in the allelic model (*I*^2^ = 50.2%; OR: 0.74; 95% CI: 0.55–1.00; *p* = 0.046) (Figure 2, Table 2). In the ApoB gene, rs693 is located in the coding region. The results showed that there was no significant relationship between this mutation and SONFH in the allelic model (*I*^2^ = 58%; OR: 2.63; 95% CI: 0.92–7.54; *p* = 0.072). However, other models showed that this mutation could increase SONFH risk (heterozygous model: *I*^2^ = 54.5%; OR: 2.46; 95% CI: 1.27–4.77; *p* = 0.008; homozygous model: *I*^2^ = 24.4%; OR: 7.70; 95% CI: 1.23–48.16; *p* = 0.029; dominant model: *I*^2^ = 31.4%; OR: 2.99; 95% CI: 1.71–5.21; *p* < 0.001; recessive model: *I*^2^ = 32.1%; OR: 7.16; 95% CI: 1.19–43.02; *p* = 0.031). rs1042031 could cause a missense mutation of glutamic acid to lysine or stop-gain mutation. Only the dominant model was analyzed, and the results showed that there was a significant relationship between this mutation and SONFH (*I*^2^ = 50.3%; OR: 2.90; 95% CI: 1.49–5.66; *p* = 0.002). Three studies were from China, and subgroup analysis results also indicated a significant relationship (*I*^2^ = 0%; OR: 4.81; 95% CI: 2.05–11.31; *p* < 0.001) (Figure 2, Table 2).

**Table 2:** Meta-analysis of the associations between SNPs and SONFH in the SNP results in more than three studies.

**Gene**	**Location**	**SNP/HWE test**	**Model**	**No. of study**	**Sample size**	**OR**	**LCI**	**UCI**	***P-*value**	***I*^2^**	***P* for *I*^2^**	**Begg'stest**	**Egger'stest**
*ABCB1*	Multinational	rs1045642	**Allelic model**	**8**	**1141**	**0.74**	**0.55**	**1.00**	**0.046**	**50.20%**	**0.02**	**0.902**	**0.797**
			Heterozygous model	8	1141	0.80	0.61	1.06	0.117	0%	0.758	1	0.891
		Case: 0.4946	Homozygous model	8	1141	0.48	0.20	1.15	0.101	67.10%	0.006	0.548	0.458
		Control: 0.0423	Dominant model	9	1420	0.78	0.61	1.00	0.051	3.80%	0.403	0.602	0.838
		Total: 0.0209	Recessive model	8	1141	0.57	0.24	1.37	0.21	71.80%	0.002	0.548	0.427
*ABCB1*	China	rs1045642	Allelic model	5	790	0.84	0.54	1.31	0.434	67.00%	0.017	0.806	0.664
			Heterozygous model	5	790	0.83	0.60	1.15	0.263	0.00%	0.803	0.806	0.427
		Case: 0.3666	Homozygous model	5	790	0.64	0.18	2.27	0.493	79.00%	0.003	1	0.934
		Control: 0.106	Dominant model	5	790	0.78	0.57	1.06	0.111	11.90%	0.338	0.462	0.518
		Total:0.0473	Recessive model	5	790	0.72	0.21	2.47	0.601	80.40%	0.002	1	0.889
*ABCB1*	Multinational	rs2032582	Allelic model	7	964	0.85	0.58	1.23	0.382	63.40%	0.012	1	0.517
			Heterozygous model	7	964	0.78	0.56	1.08	0.138	11.70%	0.34	1	0.042
		Case:0.0034	Homozygous model	7	964	0.68	0.32	1.42	0.3	60.10%	0.028	0.707	0.731
		Control:0.2588	Dominant model	7	964	0.81	0.59	1.10	0.167	39.30%	0.13	1	0.227
		Total:0.0055	Recessive model	7	964	0.85	0.45	1.63	0.633	62.20%	0.021	0.707	0.804
*ABCB1*	China	rs2032582	Allelic model	5	764	0.97	0.60	1.59	0.914	70.60%	0.009	0.806	0.408
			Heterozygous model	5	764	0.80	0.55	1.15	0.231	41.00%	0.148	1	0.077
		Case:0.0021	Homozygous model	5	764	0.85	0.32	2.24	0.742	69.70%	0.019	1	0.99
		Control:0.4073	Dominant model	5	764	0.82	0.45	1.51	0.528	57.70%	0.051	0.806	0.238
		Total:0.009	Recessive model	5	764	1.10	0.49	2.44	0.819	68.30%	0.024	1	0.87
*ApoB*	Multinational	rs693	Allelic model	4	570	2.63	0.92	7.54	0.072	58.00%	0.068	1	0.67
			**Heterozygous model**	**4**	**570**	**2.46**	**1.27**	**4.77**	**0.008**	**54.50%**	**0.111**	**1**	**0.461**
		Case:0.0027	**Homozygous model**	**4**	**570**	**7.70**	**1.23**	**48.16**	**0.029**	**24.40%**	**0.25**	**1**	**NA**
		Control:0.2173	**Dominant model**	**5**	**725**	**2.99**	**1.71**	**5.21**	<**0.001**	**31.40%**	**0.212**	**0.624**	**0.82**
		Total:0.0003	**Recessive model**	**4**	**570**	**7.16**	**1.19**	**43.02**	**0.031**	**32.10%**	**0.225**	**1**	**NA**
*ApoB*	China	rs693	Allelic model	3	415	2.82	0.51	15.57	0.235	72.20%	0.028	1	0.77
			Heterozygous model	3	415	1.59	0.32	7.86	0.57	70.20%	0.067	1	NA
		Case:0.0008	Homozygous model	3	415	16.17	0.89	293.00	0.06				
		Control:1	Dominant model	3	415	2.41	0.54	10.82	0.252	62.40%	0.07	1	0.881
		Total:0.0005	Recessive model	3	415	16.17	0.89	293.00	0.06				
*ApoB*	Multinational	rs1042031	**Dominant model**	**4**	**572**	**2.90**	**1.49**	**5.66**	**0.002**	**50.30%**	**0.11**	**0.308**	**0.146**
*ApoB*	China	rs1042031	**Dominant model**	**3**	**415**	**4.81**	**2.05**	**11.31**	<**0.001**	**0.00%**	**0.886**	**1**	**0.769**
*MTHFR*	Multinational	rs1801133	Allelic model	3	251	0.92	0.59	1.44	0.71	12.90%	0.317	1	0.401
			Heterozygous model	3	251	0.62	0.33	1.18	0.144	22.10%	0.277	1	0.549
		Case:0.0713	Homozygous model	3	251	1.24	0.48	3.22	0.653	0%	0.744	1	NA
		Control: 0.8662	Dominant model	4	507	0.94	0.61	1.45	0.775	23.40%	0.271	0.308	0.203
		Total:0.3016	Recessive model	3	251	1.54	0.63	3.75	0.339	0%	0.964	1	NA
*PAI-1*	Multinational	rs1799768	Allelic model	3	485	0.55	0.23	1.35	0.193	83.90%	0.002	0.296	0.021
			Heterozygous model	3	485	0.47	0.19	1.16	0.099	68.10%	0.043	1	0.937
		Case: 0.0016	Homozygous model	3	485	0.38	0.07	2.03	0.258	77.50%	0.012	0.296	0.069
		Control: 0.3961	Dominant model	3	485	0.44	0.14	1.34	0.146	82.80%	0.003	0.296	0.107
		Total: 0.0066	Recessive model	3	485	0.60	0.17	2.13	0.429	63.50%	0.064	0.296	0.151

*SNP, single-nucleotide polymorphism; SONFH, steroid-induced osteonecrosis of the femoral head; OR, odds ratio; LCI, lower confidence intervals; UCI, upper confidence intervals; HWE, Hardy–Weinberg equilibrium. Bold characters indicate that the p value is less than 0.05*.

**Figure 3:**
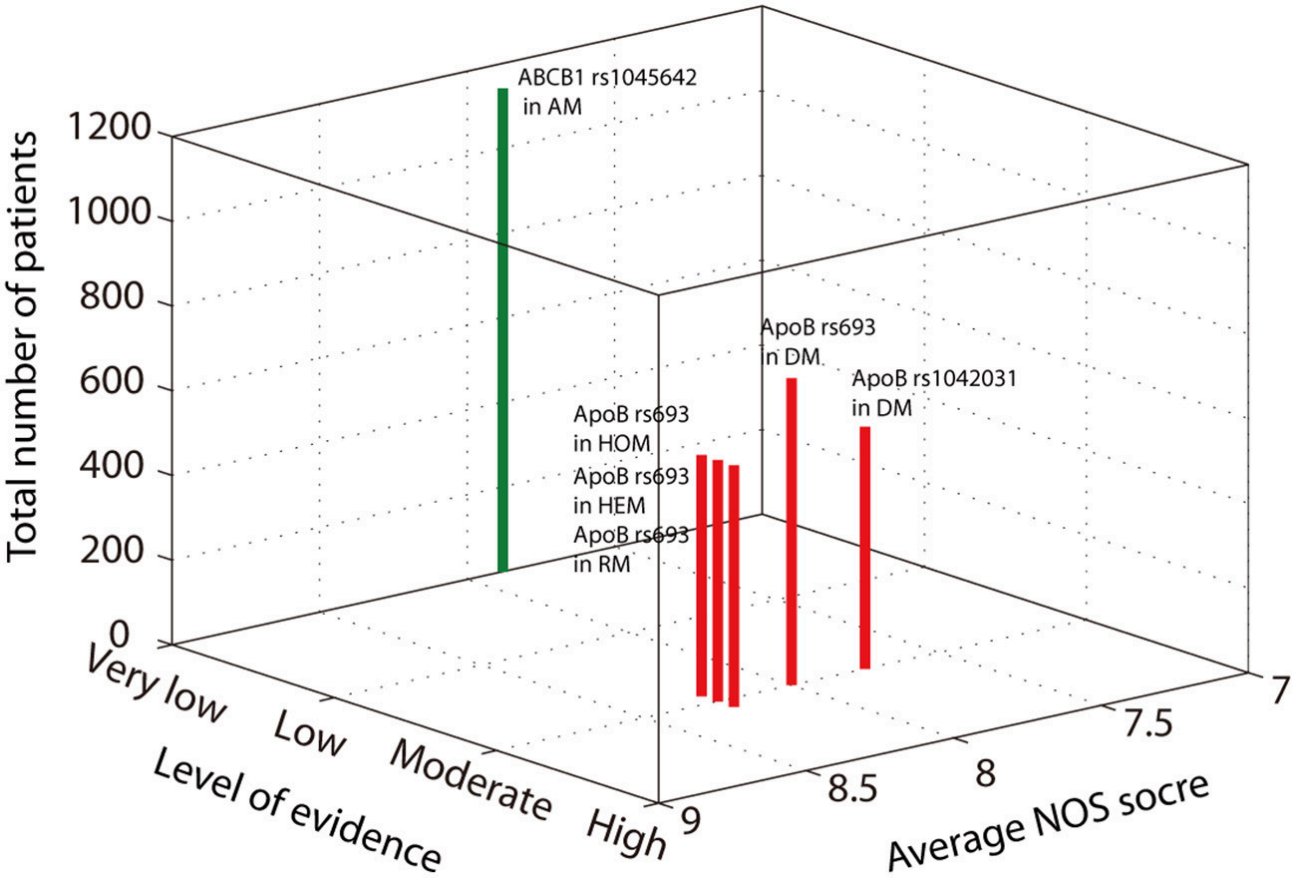
A three-dimensional Manhattan plot showing the average NOS score, the number of included patients, and the level of evidence. The green bar indicates the protection effect, and the red bar indicates the promotion effect.

The level of evidence of the above positive results was assessed according to GRADE guidelines. The allelic model of ABCB1 rs1045642 showed a protective effect against SONFH. In these results, 1,141 patients were included; the average NOS score was 7.88. Although the SE was relatively small, there was heterogeneity (*I*^2^ = 50.2%, *p* = 0.02). Thus, the level of evidence was very low (Figure 3). The heterozygous model and dominant model of ApoB rs693 showed that the mutation increases SONFH risk. The SE of the two models was smaller than 0.2, and heterogeneity was not obvious (heterozygous model: *I*^2^ = 54.5%, *p* = 0.111; dominant model: *I*^2^ = 31.40%, *p* = 0.212). The level of evidence was moderate because of the large effect (OR>2) (Figure 3). The homozygous and recessive models of ApoB rs693 also showed that the mutation increases disease risk without heterogeneity (homozygous model: *I*^2^ = 24.4%, *p* = 0.25; recessive model: *I*^2^ = 32.1%, *p* = 0.225). Although the SEs were larger than 0.2, the OR was greater than 5. Thus, the evidence levels were also moderate (Figure 3). In addition, the average NOS scores of these four models were 8 to 8.25. The dominant model of ApoB rs1042031 indicated that the mutation increases SONFH risk without heterogeneity (*I*^2^ = 50.3%, *p* = 0.11). The SE of the result was less than 0.2, and the OR was greater than 2. Thus, the level of evidence was moderate (Figure 3). However, the genetic linkage disequilibrium that exists in the above positive results might affect stability.

## Discussion

Our study analyzed the association between genetic polymorphisms and SONFH risk by comparing ONFH and non-ONFH in steroid use populations. In the SNP results of the included studies, more than three allelic models of ABCB1 rs1045642 showed that mutations protect against SONFH. The ApoB rs693 and rs1042031 mutations increase SONFH risk. According to the GRADE guidelines, the evidence levels for ApoB rs693 and rs1042031 were moderate, and ABCB1 rs1045642 was very low.

In a previous meta-analysis, Gong et al analyzed 23 studies and 35 genes and indicated that PAI-1 rs1799768 and ABCB1 rs1045642, but not MTHFR rs1801133 or ABCB1 rs2032582, were related to the risk of osteonecrosis (Gong et al., 2013). However, in this study, the control population included a healthy group and a population with other types of ONFH, which might affect the accuracy. In our study, there was no relationship between PAI-1 rs1799768 after adjusting for the control population. Guo et al assessed the correlation between SONFH and hepatic CYP3A activity and high found that CYP3A activity could reduce SONFH risk. However, the conclusion was based on animal model results, and it is unclear whether the risk is reduced in humans (Guo and Deng, 2017).

ABCB1 is a member of a superfamily of ATP-binding cassette transporters that transport variant molecules across cellular membranes. Zhou et al. researched the association between ABCB1 polymorphisms and SONFH and found that rs1045642 and rs2032582 are associated with SONFH (Zhou et al., 2015). However, this study also included a healthy population in the control population. In contrast, a Chinese review showed that rs1045642 could reduce SONFH risk, while rs2032582 is not related to SONFH (Yang J. et al., 2016). For rs1045642, our results are the same as those of a previous study, but with a very low level of evidence. The mutation does not cause changes in the amino acid sequence; thus, the specific mechanism of SONFH risk change caused by this mutation is still unknown. However, this mutation has been shown to be related to the metabolism of some drugs and to the prognosis of some cancers. This relationship may be related to differences in the characteristics of non-expressed proteins, such as codon bias, expression efficiency, and differences in mRNA characteristics. Therefore, this mutation is more suitable for clinical use as a predictor of SONFH risk for steroid users to guide individual drug application. People with the rs1045642 T allele have a low risk of SONFH with steroid application, while individuals with C allele have a high SONFH risk and should avoiding long-term high-dose steroid application, with increased duration of follow-up and frequency of diagnosis. For rs2032582, our results found that the mutation is not related to SONFH (allelic model: *p* = 0.382) with statistical heterogeneity (*I*^2^ = 63.4%, *p* = 0.012).

Additionally, we performed a *post hoc* analysis of Seth E. Karol's research that excluded SNP results with very low-level evidence (Karol et al., 2015). Then, we matched potential SNPs with GO/KEGG annotations (Table 3). According to a gene set enrichment analysis based on overrepresentation enrichment analysis, only the molecular functions of ABCB1 and ApoB showed obvious relationships with lipid transporter activity (GO: 0005319) (Zhang et al., 2005). This finding indicates that at present, SNPs involved in lipid transporter activity are more related to SONFH risk than other SNPs, which supports the lipid metabolism disorder theory. ApoB is an important factor in normal lipid metabolism, the mutation of which can also be used as a predictor of SONFH occurrence to assess the risk of SONFH and to guide steroid clinical application. Although rs693 is a synonymous variant, rs693 is related to the circulating concentration of LDL cholesterol. T allele carriers have high levels of TG, TC, and LDL-C and low HDL-C (Sandhu et al., 2008). In addition, rs1042031 is a coding sequence variant that causes glutamate to lysine mutation (C > T), or the introduction of a stop codon in the amino acid sequence (C > A). The rs1042031 mutation is important for regulating the binding of apolipoprotein B to the LDL cholesterol receptor (Liu et al., 2013). In this work, these two mutations were found to be related to SONFH. Therefore, we hypothesized that the increase of circulating LDL induced by mutation increased the risk of SONFH, supporting the hypothesis that lipid metabolism disorders are associated with SONFH. Abnormal lipid metabolism leads to bone marrow adipogenesis of the femoral head that is mainly due to hyperlipidemia and inhibition of mitochondrial dehydrogenation by steroid application. The abnormal lipid metabolism then leads to fat embolism, which blocks local blood perfusion and exacerbates local inflammation. Therefore, the question remains: does reducing circulating LDL effectively reduce the risk of SONFH with steroid application? In animal models, it has been suggested that reduced circulating lipid levels can reduce the risk of SONFH (Yang Z. et al., 2016), but the clinical effect remains to be confirmed. Additionally, in the location subgroup analysis of rs1042031 results, the heterogeneity decreased significantly from 50 to 0%. The pool results of the three Chinese studies had low heterogeneity, and the population was concentrated in Guangxi and Shandong in China (Wei, 2011a; Zeng et al., 2014; Wei et al., 2015). Another study analyzed the Japanese population (Hirata et al., 2007b). Therefore, the centralization of the included population may be the main reason for the reduction in heterogeneity.

**Table 3:** GO and KEGG annotations of possibly related genes for SONFH in this research.

**Genes**	**Gene name**	**Biological process**	**Molecular function**	**Cell component**	**KEGG**
*ABCB1^#^*	ATP-binding cassette subfamily B member 1	lipid localization;regulation of membrane lipid distribution	lipid transporter activity; drug transporter activity	Apical part of cell	ABC transporters; Bile secretion
*APOB^#^*	Apolipoprotein B	Macromolecular complex remodeling;Lipid localization;Foam cell differentiation;Protein-lipid complex subunit organization;Response to inorganic substance;Regulation of plasma lipoprotein particle levels	Lipid transporter activity; Lipoprotein particle receptor binding	Endoplasmic reticulum lumen;Protein-lipid complex;Cell body;Vesicle lumen	Vitamin digestion and absorption; Fat digestion and absorption
*BAZ2A*	Bromodomain adjacent to zinc finger domain 2A	NA	Ligand-dependent nuclear receptor binding	Transcriptional repressor complex	NA
*KLF12*	Kruppel-like factor 12	NA			
*GRIN3A*	Glutamate ionotropic receptor NMDA type subunit 3A	NA	Glutamate receptor activity; Metal ion transmembrane transporter activity; Passive transmembrane transporter activity	Transporter complex;Cell body	Nicotine addiction; Cocaine addiction; Amphetamine addiction
*COL22A1*	Collagen type XXII alpha 1 chain	NA	NA	Endoplasmic reticulum lumen;collagen trimer	NA
*FAT1*	FAT atypical cadherin 1	NA	NA	NA	NA
*CABIN1*	Calcineurin-binding protein 1	NA	Phosphatase regulator activity	Inclusion body	NA
*AMPD1*	Adenosine monophosphate deaminase 1	Cellular metabolic compound salvage	Deaminase activity; Myosin binding	NA	NA
*KCNMA1*	Potassium calcium-activated channel subfamily M alpha 1	Renal system process;Response to inorganic substance;Excretion	Metal ion transmembrane transporter activity; Passive transmembrane transporter activity	Transporter complex;Apical part of cell	Renin secretion; Insulin secretion; Salivary secretion
*PCSK5*	Proprotein convertase subtilisin/kexin type 5	Renal system process	NA	Golgi lumen	NA

# *The gene results are based on traditional meta-analysis; others are based on Genome Wide Association Study-related meta-analysis*.

### Limitations

The present study still has several limitations. First, our study was performed at the study level instead of the individual level. Second, our study did not consider the impact of primary disease or steroid therapy strategies on the results. Third, there is a large amount of heterogeneity in the results of this study. Although random effect models are used for heterogeneous results, these heterogeneities can still have a potential impact on the results. Fourth, our study only analyzed the SNPs that were examined in more than three studies, but with new research and evidence, more SNPs may be studied. Therefore, this study was limited by the research available at the time.

## Conclusion

An allelic model of ABCB1 rs1045642 showed that mutations had a protective effect against SONFH at a very low level of evidence. The mutations in ApoB rs693 and rs1042031 increased the SONFH risk with moderate levels of evidence.

## Author contributions

XC performed the conception and design of the work. XC and LZ drafted and revised the work critically. DL and JL analyzed data for work. FL and HM acquisited data for this work. All authors provide approval for publication of the content.

### Conflict of interest statement

The authors declare that the research was conducted in the absence of any commercial or financial relationships that could be construed as a potential conflict of interest.
